# Trophoblast-derived CXCL16 induces M2 macrophage polarization that in turn inactivates NK cells at the maternal–fetal interface

**DOI:** 10.1038/s41423-018-0019-x

**Published:** 2018-03-27

**Authors:** Xiao-Qiu Wang, Wen-Jie Zhou, Xin-Xin Hou, Qiang Fu, Da-Jin Li

**Affiliations:** 1 Laboratory for Reproductive Immunology, Key Laboratory of Reproduction Regulation of NPFPC, SIPPR, Shanghai Key Laboratory of Female Reproductive Endocrine Related Diseases, Hospital and Institute of Obstetrics and Gynecology, IRD, Fudan University, Shanghai Medical College, Shanghai, China; 2 College of Basic Medicine, Binzhou Medical University, Yantai, Shandong China

**Keywords:** CXCL16, trophoblasts, macrophage polarization, NK cells, maternal–fetal interface

## Abstract

Decidual macrophages (dMΦ) are distinct from the conventional macrophages present in other tissues and express M2 macrophage markers, but the molecular mechanisms of formation and the roles of M2 MΦ during pregnancy have not been completely elucidated. The crosstalk between decidual natural killer cells (dNK) and dMΦ plays an important role in the maintenance of maternal–fetal immune tolerance. Here, CXCL16 derived from first-trimester trophoblast cells induces the polarization of human M2 macrophages. The M2 MΦ polarized by CXCL16 exhibit decreased interleukin-15 production, which facilitates the inactivation of NK cells. The cytotoxicity of NK cells is attenuated by the CXCL16-polarized M2 MΦ. The data shown in the present study provide evidence to support the hypothesis that CXCL16 secreted by trophoblast cells is a key molecule involved in decidual M2 MΦ polarization, which in turn regulates the killing ability of NK cells, thereby contributing to the homeostatic and immune-tolerant milieu required for successful fetal development.

## Introduction

In a successful pregnancy, the allogeneic fetoplacental unit is not rejected by the maternal immune system, and the mechanisms involved in this process are critically important. A large and specific population of immune cells is located in the decidua. These decidual leukocytes (DLC) play important roles in local cytokine production, reducing cytotoxicity, vascularization, and placental development to maintain a healthy pregnancy.^1,2^ The number of uterine leukocytes dramatically increases during decidualization and displays an unusual composition: approximately 70% are cluster of differentiation CD56^bright^CD16^–^ natural killer (NK) cells, and the remainder include macrophages (MΦ) and T cells.^3,4^ These cells play important roles in the establishment and maintenance of maternal–fetal immune tolerance.

CXCL16 secreted by the fetus-derived trophoblasts stimulates MΦ by interacting with its receptor CXCR6 on the macrophage surface, potentially leading to the formation of a specific immune microenvironment at the maternal–fetal interface.^5^ CXCL16 is expressed as both transmembrane and soluble forms. It is a scavenger receptor for oxidized lipoproteins. CXCL16 is expressed in a variety of tissues and cells, including activated endothelial cells,^6^ Hodgkin’s disease-derived tumor cells, and MΦ.^7,8^ However, the role of CXCL16 in macrophage polarization remains unknown.

MΦ show considerable plasticity and may respond efficiently to environmental signals and change their phenotype and physiology in response to cytokines and microbial signals.^9^ These changes give rise to populations with distinct functions. MΦ are roughly categorized into types M1 and M2. The M1 phenotype is typically interleukin (IL)-12^high^ and IL-10^low^, whereas the M2 MΦ phenotype is typically IL-10^high^ and IL-12^low^. M1 MΦ are potent effector cells involved in Type 1 T helper (Th1) responses, such as cytotoxicity toward microorganisms and enhanced production of pro-inflammatory cytokines.^10,11^ In contrast, M2 MΦ suppress the inflammatory response, skew the immune response toward Th2 or regulatory IL-10-associated immunity, promote tissue remodeling, and induce angiogenesis.^12,13^

According to some supporting evidence, decidual MΦ represent alternatively activated or M2 MΦ,^14,15^ particularly since local decidual microenvironments are favorable for alternative activation. However, as shown in the study by Houser et al.,^16^ decidual MΦ secrete both anti-inflammatory M2 cytokines, such as IL-10 and transforming growth factor beta (TGF-β), and pro-inflammatory M1 cytokines, such as tumor necrosis factor (TNF) and IL-1β, and therefore cannot be strictly classified as M2 MΦ. Decidual MΦ are characterized by the expression of the mannose receptor or CD206.^17^ Only rare decidual MΦ express CD16; however, decidual MΦ express significantly higher levels of CD163^15^ than MΦ in the peripheral blood, indicating their tissue specificity.

Two NK subpopulations (CD56^bright^CD16^−^ and CD56^dim^CD16^+^) have been identified in the peripheral blood and are considered to have relatively different functions. The CD56^bright^CD16^−^ NK cells secrete larger amounts of cytokines,^18^ whereas the CD56^dim^CD16^+^ population has a stronger ability to kill tumors and virus-infected cells. The killing activity of NK cells is mediated by several activating receptors, including NKG2D and the three natural cytotoxicity receptors, NKp30, NKp44, and NKp46.^19^ NK cells also identify susceptible targets through a series of inhibitory receptors. Decidual natural killer (dNK) cells are recruited from the peripheral blood and then are educated at the maternal–fetal interface.^20,21^ Human dNK cells express high levels of CD56 and do not express CD16, thus displaying a unique transcriptional profile.^22,23^ Although dNK cells express some activating receptors and have an intact cytolytic machinery, they display poor cytotoxicity.^24,25^

The goal of this study is to clarify the roles of CXCL16 secreted by human trophoblast cells in MΦ polarization. We also set out to evaluate whether CXCL16-primed MΦ modulate NK cell activity as a possible immune tolerance mechanism at the maternal–fetal interface.

## Materials and methods

### Collection of human placental tissue and isolation and primary culture of first-trimester trophoblast cells

Villi were obtained from healthy women who underwent elective pregnancy termination (gestational age 7–9 weeks) for nonmedical causes at the Obstetrics and Gynecology Hospital of Fudan University. The study was approved by the Human Ethical Committee of the Obstetrics and Gynecology Hospital of Fudan University (Shanghai, China), and informed consent was obtained from every woman participating in the study. Tissues were immediately collected under sterile conditions into DMEM (high glucose, Invitrogen Life Technologies, CA, USA) supplemented with antibiotics (100 IU/ml penicillin and 100 g/ml streptomycin) and washed with PBS for trophoblast isolation. Briefly, the obtained placental tissue was digested with 0.25% trypsin-50,000 U/ml DNase Type I (Invitrogen Life Technologies, CA, USA) at 37 °C with gentle agitation for 10 min/cycle in four cycles. The cell suspension was then carefully layered over a discontinuous Percoll gradient (65–20%, in 5% steps) and centrifuged at 2000 r.p.m. for 20 min. The middle layer was collected and washed with DMEM-high glucose medium. Cells were diluted to a density of 5 × 10^5^ cells/ml and cultured in DMEM-high glucose complete medium (2 mM glutamine, 25 mM HEPES, 100 UI/ml penicillin, and 100 μg/ml streptomycin), supplemented with 20% heat-inactivated FBS (Gibco, NY, USA), seeded on 48-well plates precoated with Matrigel (Sigma-Aldrich, MO, USA), and incubated in a 5% CO_2_ atmosphere at 37 °C. Almost all trophoblasts isolated using this procedure were stained for cytokeratin-7, whereas few cells were stained with an anti-vimentin Ab (contaminating stromal cells). The purity of the isolated cytokeratin-7^+^ primary human trophoblast cells present in the cultures was greater than 95%. The trophoblasts we isolated here included EGFR1^+^Cyk7^+^MHC^−^ villous trophoblasts and HLA-G^+^Cyk7^+^ invasive extravillous trophoblasts. We isolated HLA-G^+^Cyk7^+^ invasive extravillous trophoblasts by flow cytometry (the purity was greater than 90%, Supplementary Figure 1) and seeded them on Matrigel-coated culture dishes. The chemokines present in the supernatant were derived from these extravillous cytotrophoblasts.

The human choriocarcinoma cell line JEG3 and trophoblast cell line HTR8 were cultured in DMEM-high glucose complete medium supplemented with 10% FBS in a 5% CO_2_ atmosphere at 37 °C.

### Isolation and culture of monocytes and NK cells

Peripheral blood samples were obtained from healthy volunteers (*n* = 25, female, aged 24–41 years; none had received medical treatment, including hormones, for at least 6 months before collection, and the subjects were recruited from the college by posters). CD14^+^ monocytes and NK cells were isolated and purified from the peripheral blood using magnetic activated cell sorter (MACS) separation kits (Miltenyi Biotec, Germany). Briefly, peripheral blood mononuclear cells (PBMCs) were isolated by density gradient centrifugation using Ficoll (Sigma-Aldrich, MO, USA) and then magnetically labeled with CD14 microbeads. The cell suspension was loaded onto an MS column (Miltenyi Biotec, Bergisch Gladbach, Germany), which was placed in the magnetic field of a MACS separator. The magnetically labeled CD14^+^ monocytes were retained within the column. The isolated CD14^+^ monocytes were fluorescently stained with fluorescein isothiocyanate (FITC)-conjugated anti-CD14 Abs (antibodies; Biolegend, CA, USA) to determine the final purity. The purity of the isolated primary human monocytes was greater than 95%. The isolated monocytes were differentiated into MΦ (monocyte-derived macrophages, MDM) by adding macrophage colony-stimulating factor (50 ng/ml, Peprotech, NJ, USA) for 6 days.

NK cells were isolated and purified by MACS negative selection. For the isolation of NK cells, the isolated monocyte-depleted PBMCs were successively incubated with a cocktail of biotin-conjugated Abs and the NK microbead cocktail. The cell suspension was applied to an MS column, which was placed in the magnetic field of a MACS separator. The unlabeled cells in the flow through represented the enriched NK cells. Isolation of highly pure NK cells was achieved by depleting magnetically labeled cells. The purity of the enriched NK cells was evaluated by flow cytometry using allophycocyanin (APC)-conjugated anti-CD56 and FITC-conjugated anti-CD3 Abs (Biolegend, CA, USA). The purity of the isolated primary human NK cells was greater than 95%.

### Enzyme-linked immunosorbent assay (ELISA) for the determination of IL-10, IL-12p70, CXCL16, and IL-15 levels

Primary trophoblast cells were seeded in 48-well plates precoated with Matrigel at a density of 5 × 10^5^ cells/well in 1 ml of medium and cultured for 96 h. The supernatant was harvested under sterile conditions for further experiments. MΦ (MDM) were seeded in 48-well plates at a density of 5 × 10^5^ cells/well. MΦ were cocultured with recombinant human CXCL16 (rhCXCL16, 100 ng/ml) (Peprotech, NJ, USA), trophoblasts, or trophoblast supernatant (50% in culture volume) in the presence or absence of anti-CXCL16 neutralizing Abs (5 μg/ml) (R&D Systems, MN, USA). The MΦ supernatant was collected at 1, 2, 3, 4, 5, and 6 days of culture. Levels of secreted IL-10, IL-12p70, and IL-15 were detected using ELISA kits, according to the manufacturer’s instructions (R&D Systems, MN, USA).

Primary trophoblast cells and JEG3 and HTR8 cells were seeded in 48-well plates at a density of 5 × 10^5^ cells/well to analyze CXCL16 production. The cell culture supernatant was collected at 1, 2, 3, and 4 days of culture. The human CXCL16 ELISA kit (R&D Systems, MN, USA) was used to measure the levels of this secreted chemokine in each supernatant according to the manufacturer’s instructions.

### Ab labeling and flow cytometry

The purity of the isolated HLA-G^+^Cyk7^+^ (APC-conjugated anti-human HLA-G, 87G, Biolegend; FITC-conjugated anti-human Cyk7, CAM5.2, BD) primary human trophoblast cells and the Ki-67 (APC-conjugated anti-human Ki-67, Biolegend) expression in NK cells were analyzed by flow cytometry. We analyzed the expression of CD14, CD80, CD86, HLA-DR, CD163, CD206, and CD274 on MΦ using flow cytometry. In the trophoblast-MΦ coculture system, both cells were seeded at a density of 5 × 10^5^ cells/well and cultured for 6 days. The fluorescent dye-conjugated Abs used in this study were FITC-conjugated anti-human CD14 (TuK4), PE Cy7-conjugated anti-human CD80 (L307.4), APC-conjugated anti-human CD86 (BU63), PE-conjugated anti-human HLA-DR (LN3), APC-conjugated anti-human CD206 (19.2), PE-conjugated anti-human CD163 (GHI/61), and PE-conjugated anti-human CD274 (MIH1). Mouse IgG2a, IgG2b, and IgG1 immunoglobulins conjugated with the respective fluorochromes served as isotype controls.

NK cells were cultured alone or cocultured with MΦ that had been pretreated with (rhMΦ) or without (MΦ) 100 ng/ml rhCXCL16 for 5–6 days, and both cells were seeded at a density of 5 × 10^5^ cells/well and cultured for 6 days. NK cells were cocultured directly with MΦ or rhMΦ in the presence or absence of recombinant human IL (rhIL)-15 (10 ng/ml); both cells were seeded at a density of 5 ×1 0^5^ cells/well and cultured for 6 days. The expression of CD56, CD16, NKp30, NKp44, NKp46, TNF, IFN-γ, KIR2DL1, and KIR3DL1 on NK cells was analyzed using flow cytometry. The fluorescent dye-conjugated Abs used for this experiment were APC-conjugated anti-human CD56 (CMSSB), FITC-conjugated anti-human CD16 (CB16), PE-conjugated anti-human NKp30 (AF29-4D12), PE-conjugated anti-human NKp44 (44.189), PE Cy7-conjugated anti-human NKp46 (9E2), PE-conjugated anti-human TNF (MAb11), PE-conjugated anti-human IFN-γ (4S.B3), PE-conjugated anti-human KIR2DL1 (HP-MA4), and PE-conjugated anti-human KIR3DL1 (DX9). Mouse IgG2a, IgG2b, and IgG1 immunoglobulins conjugated with the respective fluorochromes served as isotype controls. All Abs were purchased from eBioscience, except for CD80 and KIR3DL1 (purchased from BD Biosciences).

MΦ or NK cells were blocked with 10% normal horse serum (Sigma-Aldrich, MO, USA) at room temperature for 15 min before staining. Cells were incubated with the fluorescent dye-conjugated Abs and isotype-matched controls at the recommended dilutions for 30 min in the dark. For TNF and IFN-γ staining, 1 ml of 1X Fix/Perm solution (BioLegend) was added to each tube, vortexed, and incubated at room temperature for 20 min. Cells were then centrifuged to remove the supernatant. Cells were resuspended in 1 ml of 1X Perm buffer (BioLegend) and incubated at room temperature for 15 min. Cells were centrifuged and the supernatant was discarded. Appropriate amounts of fluorochrome-conjugated anti-TNF and anti-IFN-γ Abs were then added to the resuspended cells and incubated for 30 min in the dark. Cells were then washed once with 1 ml of PBS by centrifugation at 1500 r.p.m. for 8 min and analyzed using a FACSCalibur flow cytometer and CellQuest software (BD Biosciences, NJ, USA). For double labeling, NK cells (CD56-positive) were gated and the expression of CD16, NKp30, NKp44, NKp46, TNF, IFN-γ, KIR2DL1, and KIR3DL1 was analyzed.

### Cytotoxicity assay

NK cell-mediated cytotoxicity was determined using the lactate dehydrogenase (LDH) release assay. The level of LDH released into the medium was assessed using a cytotoxicity detection kit (Cayman Chemical, MI, USA) to reflect cell death. Target cells (JEG3, HTR8 or primary trophoblast cells) were seeded into 96-well flat bottom plates at a density of 1 × 10^5^ cells per well. NK cells were cocultured with rhCXCL16-untreated or -treated MΦ in the presence or absence of 10 ng/ml rhIL-15. MΦ were cocultured with NK cells; both cells were plated at a density of 5 × 10^5^ cells/well. After 6 days, NK cells were separated from the coculture units with Miltenyi microbeads and then cocultured directly with JEG3, HTR8, or primary trophoblast cells at effector/target ratios of 5:1. Cell mixtures were then incubated for 48 h. LDH activity was measured according to the manufacturer’s instructions. Absorbance was monitored at 490 nm to quantify LDH concentrations. Experiments were performed in triplicate and repeated three times. The expression of CD107a (PE Cy7-conjugated anti-human CD107a, H4A3, Biolegend) in NK cells was detected by flow cytometry.

### Statistical analysis

All values are presented as the means ± SE. Data were analyzed using a paired-sample *t*-test or one-way analysis of variance. Statistical calculations were performed using computer software (Social Sciences software version 11.5). Differences were accepted as significant at *P *< 0.05.

## Results

### Primary first-trimester trophoblast cells secrete CXCL16

**Fig. 1:**
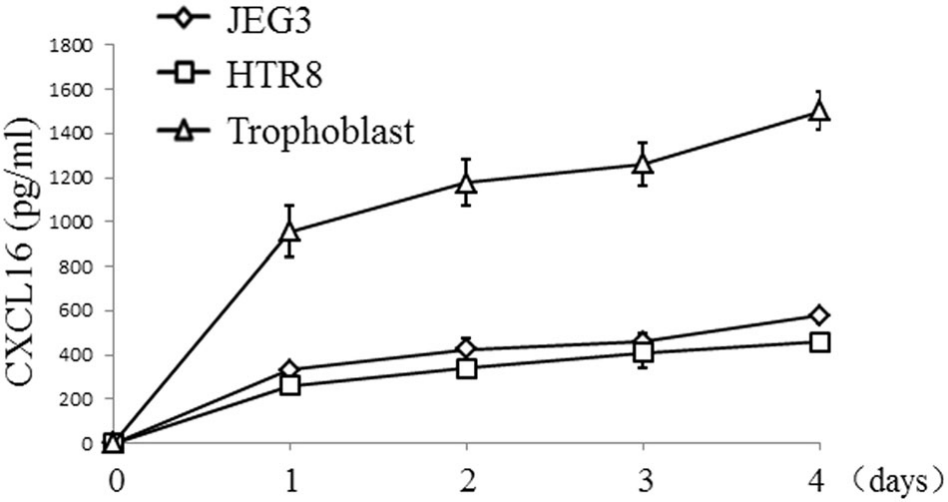
Primary human trophoblast cells and JEG3 and HTR8 cell lines secrete CXCL16. Isolated primary human trophoblast cells, the human choriocarcinoma cell line JEG3 and the trophoblast cell line HTR8 were cultured in 48-well plates at a density of 5 × 10^5^ cells/well in 1 ml of media for 24–96 h. We determined the concentration of soluble CXCL16 in culture supernatant using an ELISA at 1, 2, 3, and 4 days

Primary human trophoblast cells display a weak proliferative ability and a short survival period in vitro. They survive for more than 1 week when they are seeded on Matrigel-coated plates. We cultured isolated trophoblast cells, as well as the human choriocarcinoma cell line JEG3 and the trophoblast cell line HTR8, in Matrigel-precoated plates for 1–4 days. The release of soluble CXCL16 in the supernatant was examined by ELISA daily. Primary trophoblast cells and JEG3 and HTR8 cell lines secreted CXCL16 in a time-dependent manner (Fig. 1).

### CXCL16 induces M2 MΦ polarization

**Fig. 2:**
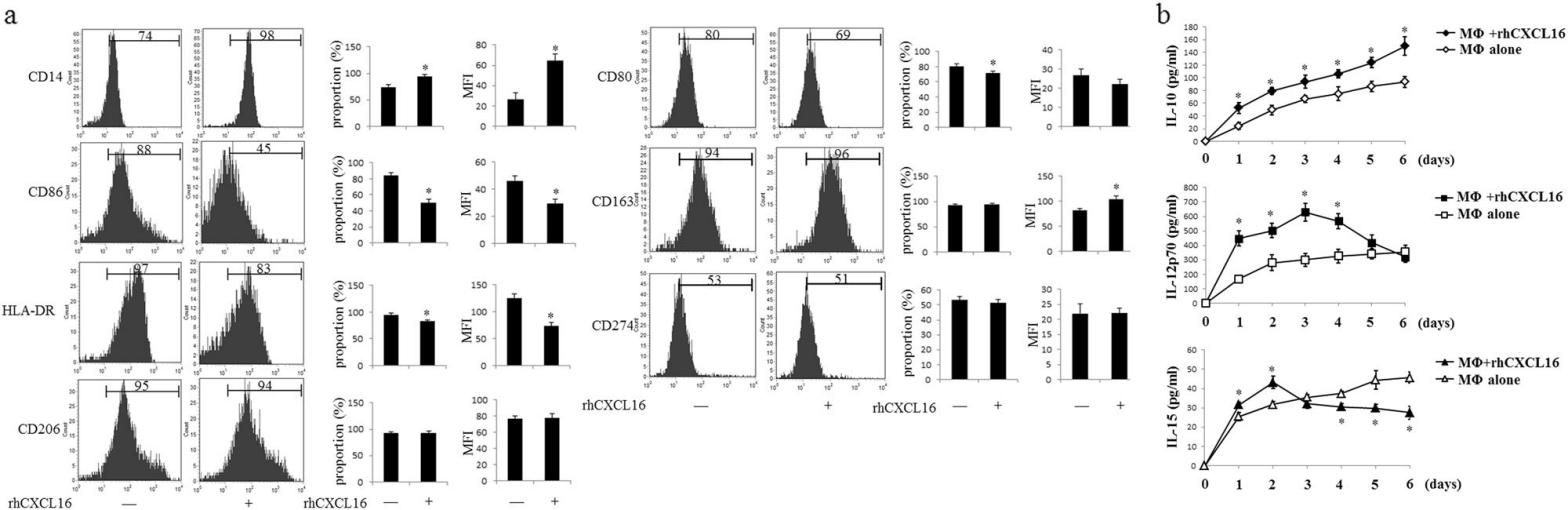
CXCL16 induces the polarization of macrophages toward the M2 phenotype. Primary human MΦ were treated with rhCXCL16 (100 ng/ml) for 6–7 days. The expression of macrophage phenotype markers, including CD14, CD80, CD86, HLA-DR, CD163, CD206, and CD274, were analyzed by flow cytometry (**a**). The concentrations of the anti-inflammatory cytokine IL-10, pro-inflammatory cytokine IL-12p70, and IL-15 in the supernatant were measured at 1, 2, 3, 4, 5, and 6 days using ELISAs (**b**). The results were based on three repeated experiments (*P < 0.05 compared to MΦ that were not treated with rhCXCL16)

MΦ may be shaped by the tissues in which they reside, and they are able to change their functions in response to different microenvironments, forming a broad repertoire of MΦ functions.^26^ Consistent with our previous findings,^5^ fetus-derived trophoblasts secreted large amounts of CXCL16 in the present study. We treated the primary human MΦ (obtained from peripheral blood) with rhCXCL16. The rhCXCL16 treatment upregulated the expression of CD14 and CD163 and downregulated the expression of CD80, CD86, and HLA-DR on MΦ (Fig. 2a). The rhCXCL16 treatment also increased IL-10 production in MΦ. Although rhCXCL16 enhanced IL-12p70 secretion within the first 3 days, its production subsequently decreased. The rhCXCL16 treatment decreased IL-15 secretion from MΦ after an initial increase (Fig. 2b). Based on these results, CXCL16 may play a critical role in the polarization of MΦ toward the M2-like phenotype by inducing high IL-10 expression.

### Primary trophoblast cells induce M2 MΦ polarization by secreting CXCL16

**Fig. 3:**
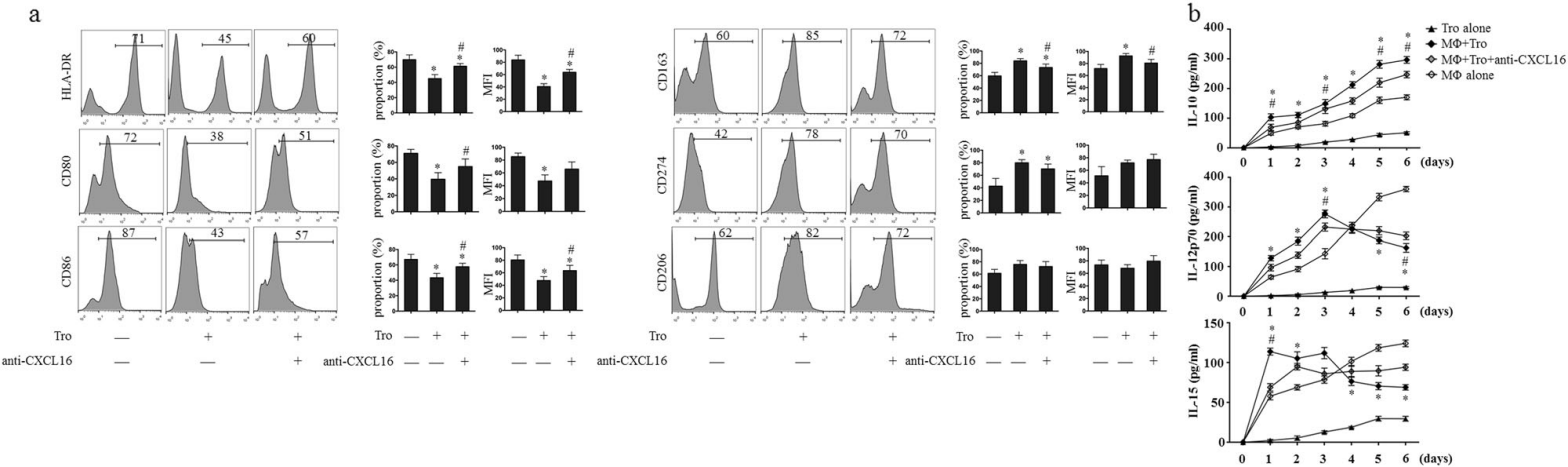
Primary trophoblast cells induce M2 macrophage polarization by secreting CXCL16. MΦ were cultured alone or cocultured with trophoblast cells in the presence or absence of the anti-CXCL16 neutralizing antibody. The expression of phenotypic markers (CD80, CD86, HLA-DR, CD163, CD274, and CD206) on MΦ was analyzed by FCM (**a**) (*P < 0.05 compared to MΦ cultured alone, # *P *< 0.05 compared to MΦ treated with trophoblast cell supernatants.). The concentrations of IL-10, IL-12p70, and IL-15 in the supernatants were determined at 1, 2, 3, 4, 5, and 6 days (**b**) (*P < 0.05 compared to MΦ cultured alone, # *P *< 0.05 compared to MΦ treated with trophoblast cell supernatant supplemented with the anti-CXCL16 neutralizing antibody.) The results were based on three repeated experiments

We next investigated whether the CXCL16 secreted by primary trophoblast cells would induce the expression of M2 macrophage phenotypic markers. MΦ (obtained from peripheral blood) were cocultured with trophoblast cells in the presence or absence of anti-CXCL16 neutralizing Abs. The expression of phenotypic markers on MΦ was analyzed. The expression of CD163 and CD274 was upregulated, whereas the expression of CD80, CD86, and HLA-DR was downregulated, and the anti-CXCL16 neutralizing antibody treatment partially reversed these effects (Fig. 3a). Trophoblast cells significantly promoted IL-10 production in MΦ. Although trophoblast cells stimulated MΦ to secrete IL-12p70 within the first 3 days, these cells subsequently inhibited IL-12p70 production. Trophoblast cells decreased IL-15 secretion from MΦ after the initial increase observed within the first 3 days. The treatment with anti-CXCL16 neutralizing Abs partially blocked the effect of trophoblast cells on MΦ (Fig. 3b). Thus, primary trophoblast cells induce macrophage polarization by secreting CXCL16.

**Fig. 4:**
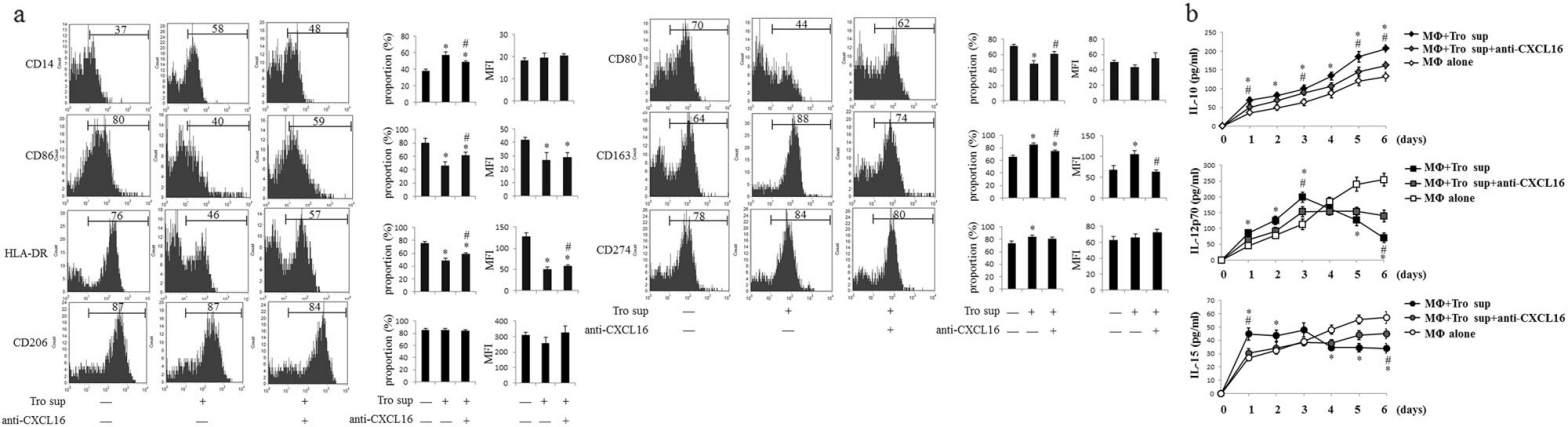
CXCL16 secreted by primary trophoblast cells induces the polarization of M2 macrophages. MΦ were cultured alone or treated with trophoblast cell supernatants in the presence or absence of the anti-CXCL16 neutralizing antibody. The expression of phenotypic markers (CD14, CD80, CD86, HLA-DR, CD163, CD274, and CD206) on MΦ was analyzed by FCM (**a**) (*P < 0.05 compared to MΦ cultured alone, # *P *< 0.05 compared to MΦ treated with trophoblast cell supernatants.). The concentrations of IL-10, IL-12p70, and IL-15 in the supernatants were determined at 1, 2, 3, 4, 5, and 6 days (**b**) (*P < 0.05 compared to MΦ cultured alone, #*P *< 0.05 compared to MΦ treated with trophoblast cell supernatant supplemented with the anti-CXCL16 neutralizing antibody). The results were based on three repeated experiments

MΦ (obtained from peripheral blood) were treated with trophoblast cell supernatants from trophoblast cells cultured with or without anti-CXCL16 neutralizing Abs to further confirm the role of CXCL16 secreted by primary trophoblast cells on the expression of M2 macrophage phenotypic markers. The expression of phenotypic markers on MΦ were analyzed. The expression of CD14, CD163, and CD274 was upregulated, whereas the expression of CD80, CD86, and HLA-DR was downregulated, and the anti-CXCL16 neutralizing antibody treatment partially reversed these effects (Fig. 4a) The trophoblast cell supernatant promoted IL-10 production in MΦ. Although the trophoblast cell supernatant stimulated MΦ to secrete IL-12p70 within the first 3 days, it subsequently inhibited IL-12p70 production. Treatment with the trophoblast cell supernatant decreased IL-15 secretion from MΦ after the initial increase observed in the first 3 days. Treatment with anti-CXCL16 neutralizing Abs partially blocked the effect of the trophoblast cell supernatant on MΦ (Fig. 4b). Therefore, CXCL16 secreted by primary trophoblast cells participates in establishing macrophage plasticity.

### CXCL16-polarized M2 MΦ facilitate the inactivation of NK cells

**Fig. 5:**
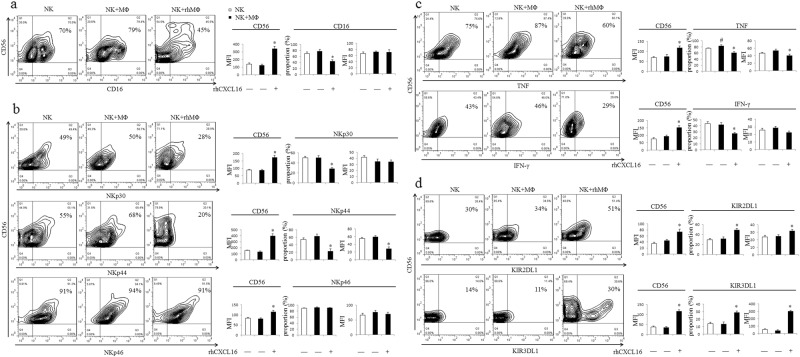
CXCL16-polarized M2 MΦ induce NK inactivation. Primary NK cells were cultured alone or cocultured with MΦ that had been pretreated with or without rhCXCL16. The expression of CD56, CD16 (**a**), NKp30, NKp44, NKp46 (**b**), TNF, IFN-γ (**c**), KIR2DL1 and KIR3DL1 (**d**) in NK cells was analyzed by FCM (*P < 0.05 compared to NK cells cultured alone and/or cocultured with MΦ, #*P *< 0.05 compared to NK cells cultured alone)

We examined whether M2 MΦ polarized by CXCL16 influenced NK cell activation to further clarify the role of CXCL16-polarized MΦ in maternal–fetal immune tolerance. We cocultured primary NK cells (obtained from peripheral blood) with MΦ (obtained from peripheral blood) that had been pretreated with or without rhCXCL16. MΦ that had not been pretreated with rhCXCL16 had little effect on the activation of NK cells, except for upregulating TNF production in NK cells. In contrast, the M2 MΦ polarized by rhCXCL16 clearly upregulated CD56 expression on NK cells. The rhCXCL16-polarized M2 MΦ not only downregulated the expression of the activating receptors NKp30 and NKp44 and decreased the production of TNF and IFN-γ but also upregulated the expression of the inhibitory receptors KIR2DL1 and KIR3DL1 on NK cells (Fig. 5a–d). Therefore, M2 MΦ polarized by CXCL16 induce a phenotype compatible with reduced NK cell cytotoxicity. Since CXCL16 secreted by trophoblast cells inhibited IL-15 production in MΦ after an initial stimulatory effect, we speculated that M2 MΦ polarized by CXCL16 may modulate the phenotype of NK cells by decreasing IL-15 expression.

### CXCL16-polarized M2 MΦ induce NK cell inactivation by decreasing IL-15 production

**Fig. 6:**
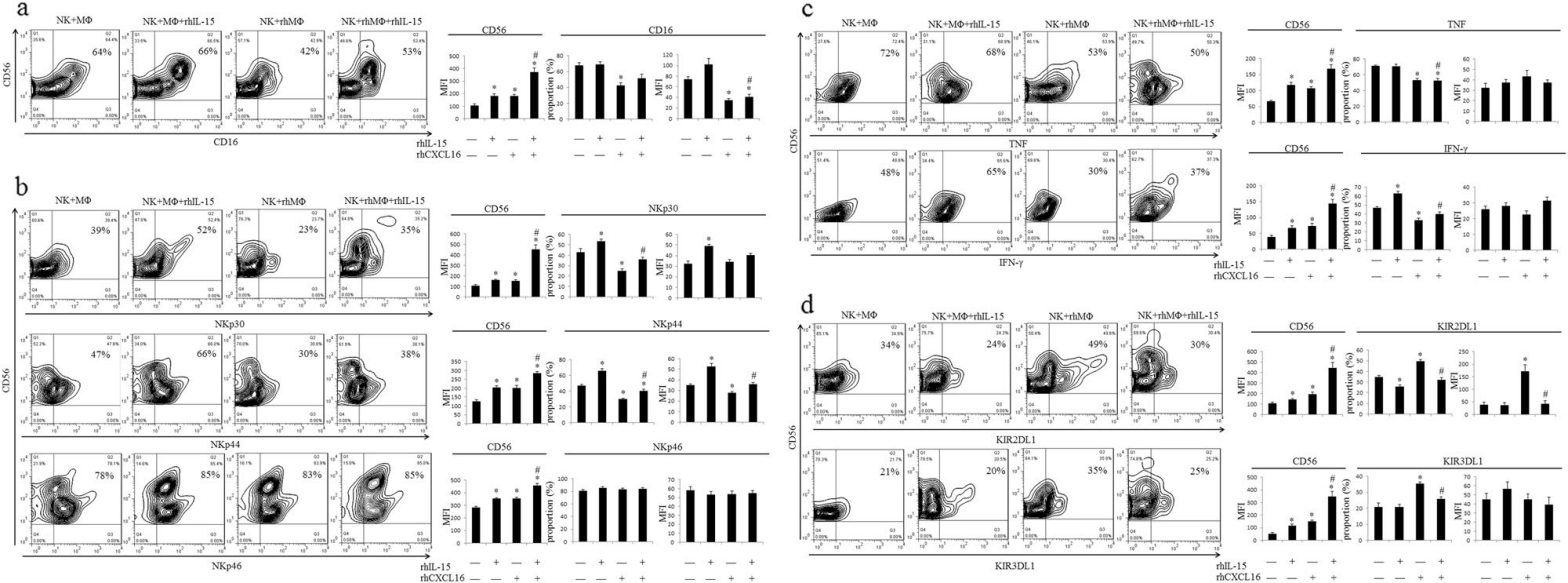
M2 MΦ polarized by CXCL16 induce the inactivation of NK cells by decreasing IL-15 expression. NK cells were cocultured with MΦ pretreated with or without rhCXCL16; rhIL-15 was added to some groups. The expression of CD56, CD16 (**a**), NKp30, NKp44, NKp46 (**b**), TNF, IFN-γ (**c**), KIR2DL1 and KIR3DL1 (**d**) in NK cells was detected by FCM. The results were obtained from three individual experiments (*P < 0.05 compared to NK cells cocultured with MΦ, #*P *< 0.05 compared to NK cells cocultured with MΦ that had been polarized by rhCXCL16 and/or NK cells cocultured with MΦ treated with rhIL-15)

Next, we investigated whether the M2 MΦ polarized by CXCL16 regulated NK cell function by modulating IL-15 expression. NK cells (obtained from peripheral blood) were cocultured with MΦ (obtained from peripheral blood) that had been pretreated with or without rhCXCL16, and rhIL-15 was added to some groups. As shown in Fig. 6, NK cells cocultured with MΦ pretreated with rhCXCL16 or cocultured with the MΦ supplemented with exogenous IL-15 showed increased expression of CD56, and NK cells cocultured with MΦ pretreated with rhCXCL16 supplemented with rhIL-15 showed even higher CD56 expression. M2 MΦ polarized by CXCL16 downregulated the expression of CD16 (a), NKp30, and NKp44 (b), TNF and IFN-γ (c), and upregulated the expression of KIR2DL1 and KIR3DL1 on NK cells (d). These effects were partially blocked by supplementing the cells with rhIL-15. NK cells cocultured with MΦ supplemented with exogenous IL-15 displayed increased NKp30, NKp44, and IFN-γ expression, and decreased KIR2DL1 expression compared with NK cells cocultured with MΦ. Based on these results, CXCL16 produced by trophoblast cells plays a pivotal role in inducing the generation of M2 MΦ, which are characterized by a decrease in IL-15 production and subsequent NK cell inactivation. We detected Ki-67 expression in NK cells using FCM to analyze NK cell viability (Supplementary Figure 2) and found that NK cells exhibit a slight increase (1.18 fold) in viability (Ki-67 expression) when cocultured with MΦ supplemented with IL-15 compared to NK cells cultured alone. Therefore, the differences in cytotoxicity and cytokine secretion between the groups were mainly due to the cell activation state.

### NK cytotoxicity is attenuated by CXCL16-polarized MΦ

**Fig. 7:**
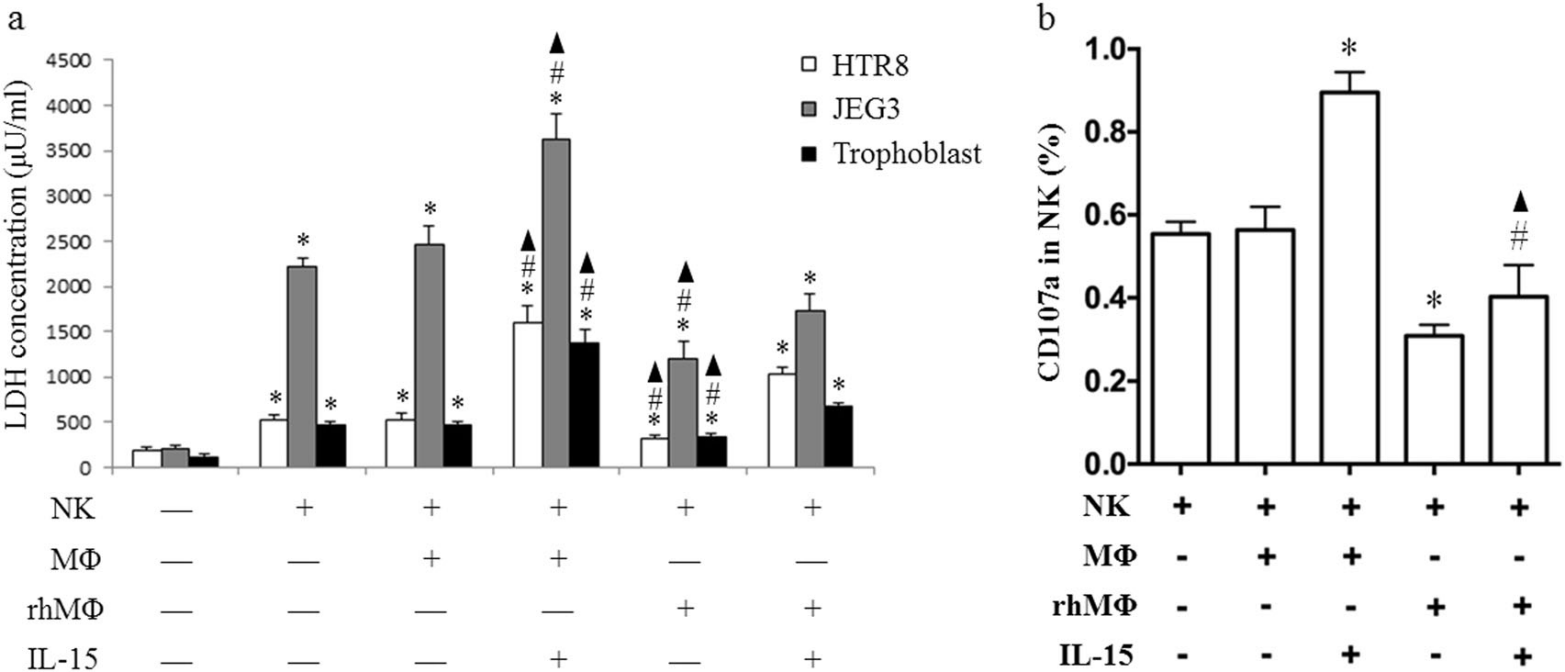
CXCL16-polarized M2 MΦ attenuate the cytotoxicity of NK cells. Primary human NK cells were cultured alone or cocultured with MΦ that had been pretreated with or without rhCXCL16, and rhIL-15 was added to some coculture groups. NK cells were then isolated and cocultured with primary human trophoblast cells or the JEG3 or HTR8 cell lines. The cytotoxicity of NK cells was detected using the LDH release assay (*P < 0.05 compared to the control in the same group, #*P* < 0.05 compared to NK cells cocultured with MΦ, ▲*P* < 0.05 compared to NK cells cocultured with MΦ that had been polarized by rhCXCL16 and treated with rhIL-15.) (**a**) and by measuring CD107a expression (**b**) (*P < 0.05 compared to NK cells cocultured with MΦ, #*P* < 0.05 compared to NK cells cocultured with MΦ treated with rhIL-15, ▲*P* < 0.05 compared to NK cells cocultured with MΦ that had been polarized by rhCXCL16)

**Fig. 8:**
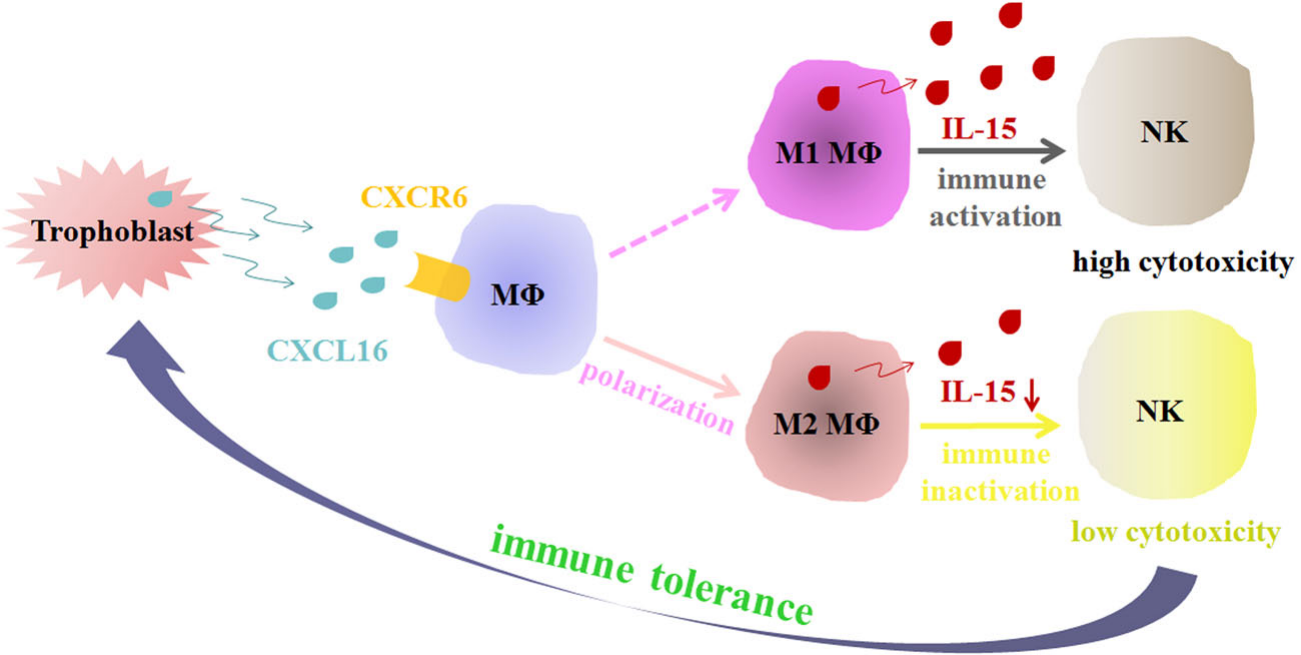
Schematic diagram of the data presented in this article. At the maternal–fetal interface, CXCL16 secreted by trophoblast cells induces the polarization of macrophages toward the M2 phenotype. M2 MΦ polarized by CXCL16 attenuate the cytotoxicity of NK cells by decreasing IL-15 secretion, which contributes to the formation of maternal–fetal immune tolerance

Primary NK cells (obtained from peripheral blood) were cultured alone or cocultured with MΦ (obtained from peripheral blood) pretreated with or without rhCXCL16 to evaluate the cytotoxicity of NK cells primed by MΦ with different polarization states; rhIL-15 was added to some coculture groups. NK cells were then detected by FCM or were isolated and cocultured with primary human trophoblast cells or JEG3 or HTR8 cell lines. NK cells cocultured with MΦ supplemented with exogenous IL-15 displayed a significant increase in the LDH concentration. NK cells cocultured with MΦ polarized by rhCXCL16 displayed a decreased LDH concentration compared with NK cells cocultured with MΦ, and rhIL-15 reversed this effect. Similar results were obtained in primary trophoblast cells and JEG3 and HTR8 cell lines (Fig. 7a). Moreover, NK cells cocultured with MΦ supplemented with exogenous IL-15 displayed a significant increase in CD107a expression. NK cells cocultured with MΦ polarized by rhCXCL16 exhibited decreased CD107a expression compared with NK cells cocultured with MΦ, and rhIL-15 partially reversed this effect (Fig. 7b). According to the results of the cytotoxicity assays, CXCL16 secreted by trophoblasts regulated maternal MΦ to display M2 MΦ functional behaviors by decreasing IL-15 secretion from cells at the maternal–fetal interface, which in turn modulated the responses of NK cells in this critical location to maintain a healthy pregnancy (Fig. 8).

## Discussion

One of the ways in which the maternal immune system addresses the challenge of tolerating the presence of the allogeneic fetus is the unique distribution of DLC. A substantial increase in the numbers of different leukocytes is observed in the first-trimester decidua.^27^ The most abundant leukocytes are NK cells, MΦ, and T cells. These various cells interact and play pivotal roles in placentation and fetal development.^28^ In the present study, we investigated the role of CXCL16 in MΦ polarization to provide insights that will enable a better understanding of the progression of MΦ polarization at the maternal–fetal interface.

The fetal cytotrophoblast is a key placental cell that plays a crucial role in a successful pregnancy. During early pregnancy, human trophoblasts continuously produce large amounts of CXCL16.^5^ As shown in our previous studies, first-trimester human cytotrophoblasts co-express CXCL16 (transmembrane and soluble forms) and CXCR6, which promotes their proliferation and invasion in an autocrine manner.^29^ CXCR6 is selectively expressed in leukocytes, particularly T lymphocytes and CD14^+^ monocytes. NK cells express little CXCR6 at the human maternofetal interface. T lymphocytes and monocytes migrate into the decidua by expressing CXCR6.^5^

CXCL16 secreted by human trophoblast cells may play an important role in inducing M2 MΦ polarization. CXCL16 upregulates the expression of CD14, CD163, and CD274, and downregulates the expression of CD80, CD86, and HLA-DR on MΦ (the MΦ were obtained from peripheral blood). The environmental signals (cytokines, growth factors, and microorganism-associated molecular patterns) to which the monocytes-MΦ are exposed define their phenotypes and functions, with the two extremes being the classic M1 MΦ and the alternative M2 MΦ.^30,31^ Determination of the molecular mechanisms underlying MΦ polarization is a very active research area, which will provide novel opportunities for manipulating immune and inflammatory responses. The mechanism by which M2 MΦ tune the inflammatory responses is through the production of anti-inflammatory cytokines, such as IL-10, TGF-β, and PGE2. Consistent with this finding, M2 MΦ polarized by CXCL16 exhibit increased expression of IL-10 and decreased expression of IL-12p70.

In humans, dMΦ display an M2 phenotype and may be involved in remodeling the endometrium, trophoblast invasion, and the development of the tolerant milieu required for successful progression of pregnancy.^14,15,17,32,–35^ According to the results from previous studies, dMΦ express CD163, CD206, and CD209,^15^ and low levels of the costimulatory molecules CD80 and CD86. Moreover, M-CSF and IL-10, both of which are produced by placental trophoblast cells,^36^ induce an M2 phenotype similar to dMΦ. However, researchers have not determined whether chemokines contribute to this process. Here, we identified a new mechanism for the polarization of dMΦ. Based on the phenotype and cytokine secretion patterns, CXCL16 is a potent inducer of M2-like MΦ, which may, therefore, play a critical role in regulating decidual MΦ in early pregnancy. CXCL16 decreases IL-12p70 production in MΦ after the initial upregulation, consistent with MΦ first passing through an inflammatory “M1-like” phase before transitioning to an alternative activation phenotype.

The dNK may regulate angiogenesis and placental developmental processes, such as growth, differentiation, and invasion of trophoblastic cells.^37^ These cells are conducive to tissue remodeling and secrete various growth factors that contribute to the implantation of the embryo.^18,38^ Accordingly, defects in dNK generation and/or function may be responsible for fetal loss.

In the presence of rhCXCL16-treated MΦ, the cytotoxicity of NK cells obtained from peripheral blood is attenuated. NK cells and MΦ regulate each other. Cytokines, such as IL-15 and IL-12, produced by activated MΦ play important roles in regulating NK activity.^39^ The crosstalk between MΦ and NK cells plays important roles in antitumor and anti-infection responses.^40,41^ Remarkably, a histochemical analysis has revealed that dNK cells are in close contact with dMΦ,^42^ suggesting potential functional crosstalk between these two cell types.^18,43^ The crosstalk between dNK cells and dMΦ might result in the selective expansion of Tregs.^44^ Furthermore, Tregs are considered important cells that prevent the generation of maternal alloreactive T cells thought to be a major cause of immune-mediated miscarriages.^45^ Therefore, the interaction between NK cells and MΦ may be involved in shaping key features of the placenta, such as the maintenance of immune suppression in the placental microenvironment.

CXCL16 decreases IL-15 expression in MΦ. IL-15 plays important roles in the differentiation, maturation, and survival of NK cells.^46,47^ IL-15 induces the expression of a chemokine receptor pattern in pNK cells that is similar to uterine NK (uNK) cells.^48^ The phenotype of endometrial NK cells resembles the phenotype of dNK cells after activation with IL-15.^49^ IL-15 is a key cytokine required for the differentiation of hematopoietic progenitors to NK cells, and its expression in the decidua increases after conception.^50^ IL-15, which is secreted by MΦ, is an important cytokine that regulates the growth, proliferation,^51^ differentiation, and expression of cytotoxic molecules in decidual NK cells. It also promotes the development of the cytotoxic activity of NK cells in vitro.^52^ In very early pregnancy, the increase in IL-15 production by MΦ may support desirable mild pro-inflammatory reactions, increase NK cell numbers, and their cytotoxic potential, leading to better trophoblast growth control. Subsequently, the M2 dMΦ may impair NK cytotoxicity by decreasing IL-15 secretion to establish immune tolerance.

Based on our results, CXCL16 secreted by human trophoblasts contributes to MΦ polarization and shapes the anti-inflammatory behavior of MΦ, which may restrict the cytotoxicity of NK cells. To our knowledge, this is the first report describing the relevance of CXCL16 in human MΦ polarization and its involvement in the acquisition of the phenotype and effector functions commonly associated with M2/anti-inflammatory MΦ. Therefore, strategies targeting molecules such as CXCL16 that support M2-type polarization are likely to provide therapeutic benefits in preventing pregnancy failure. However, the findings in the present study were obtained using in vitro models. Further research is needed to determine whether CXCL16 skews MΦ toward an M2 phenotype in vivo and the regulatory mechanism involved. Further studies of the molecular mechanisms underlying MΦ polarization may improve our understanding of pregnancy-associated diseases.

## Electronic supplementary material

Supplementary Figure 1

Supplementary Figure 2

## References

[1] Croy BA, Chantakru S, Esadeg S, Ashkar AA, Wei QX (2002). Decidual natural killer cells: key regulators of placental development. J. Reprod. Immunol..

[2] Boyson JE (2002). CD1d and invariant NKT cells at the human maternal-fetal interface. Proc. Natl Acad. Sci. USA.

[3] Bulmer JN, Morrison L, Longfellow M, Rittson A, Pace D (1991). Granulated lymphocytes in human endometrium-histochemical and immunohistochemical studies. Hum. Reprod..

[4] Starkey PM, Sargent IL, Redman CW (1988). Cell-populations in human early-pregnancy decidua-characterization and isolation of large granular lymphocytes by flow cytometry. Immunology.

[5] Huang Y, Zhu XY, Du MR, Li DJ (2008). Human trophoblasts recruited T lymphocytes and monocytes into decidua by secretion ofchemokine CXCL16 and interaction with CXCR6 in the first-trimester pregnancy. J. Immunol..

[6] Volin MV (2001). Fractalkine: a novel angiogenic chemokine in rheumatoid arthritis. Am. J. Pathol..

[7] Hanamoto H (2004). Expression of CCL28 by Reed–Sternberg cells defines a major subtype of classical Hodgkin’s disease with frequent infiltration of eosinophils and/or plasma cells. Am. J. Pathol..

[8] Wagsater D, Hugander A, Dimberg J (2004). Expression of CXCL16 in human rectal cancer. Int J. Mol. Med..

[9] Mosser DM, Zhang X (2008). Interleukin-10: new perspectives on an old cytokine. Immunol. Rev..

[10] Gordon S (2003). Alternative activation of macrophages. Nat. Rev. Immunol..

[11] Mantovani A (2004). The chemokine system in diverse forms of macrophage activation and polarization. Trends Immunol..

[12] Svensson-Arvelund J, Ernerudh J (2015). The role of macrophages in promoting and maintaining homeostasis at the fetal–maternal interface. Am. J. Reprod. Immunol..

[13] Lash GE (2016). Decidual macrophages: key regulators of vascular remodeling in human pregnancy. J. Leukoc. Biol..

[14] Gustafsson C (2008). Gene expression profiling of human decidual macrophages: evidence for immunosuppressive phenotype. PLoS ONE.

[15] Svensson J (2011). Macrophages at the fetal–maternal interface express markers of alternative activation and are induced by M-CSF and IL-10. J. Immunol..

[16] Houser BL, Tilburgs T, Hill J, Nicotra ML, Strominger JL (2011). Two unique human decidual macrophage populations. J. Immunol..

[17] Laskarin G (2005). The presence of functional mannose receptor on macrophages at the maternal–fetal interface. Hum. Reprod..

[18] Hanna J (2006). Decidual NK cells regulate key developmental processes at the human fetal–maternal interface. Nat. Med..

[19] Arnon TI, Markel G, Mandelboim O (2006). Tumor and viral recognition by natural killer cells receptors. Semin Cancer Biol..

[20] Li YH (2016). The Galectin-9/Tim-3 pathway is involved in the regulation of NK cell function at the maternal-fetal interface in early pregnancy. Cell Mol. Immunol..

[21] Li, Y., Li, D., Du, M. TIM-3: the crucial regulator of NK cells in pregnancy. *Cell. Mol. Immunol*. (2017), in press. 10.1038/cmi.2017.85 PMC5675961 28890545

[22] Koopman LA (2003). Human decidual natural killer cells are a unique NK cell subset with immunomodulatory potential. J. Exp. Med..

[23] Tao Y (2015). CD56(bright) CD25(+) NK cells are preferentially recruited to the maternal/fetal interface in early human pregnancy. Cell. Mol. Immunol..

[24] Tabiasco J (2006). Human decidual NK cells: unique phenotype and functional properties: a review. Placenta.

[25] Kopcow HD (2005). Human decidual NK cells form immature activating synapses and are not cytotoxic. Proc. Natl Acad. Sci. USA.

[26] Stout RD, Suttles J (2004). Functional plasticity of macrophages: reversible adaptation to changing microenvironments. J. Leukoc. Biol..

[27] Strominger JL (2004). Human decidual lymphocytes and the immunobiology of pregnancy. J. Reprod. Immunol..

[28] von Rango U (2008). Fetal tolerance in human pregnancy: a crucial balance between acceptance and limitation of trophoblast invasion. Immunol. Lett..

[29] Huang Y (2006). Chemokine CXCL16, a scavenger receptor, induces proliferation and invasion of first-trimester human trophoblast cells in an autocrine manner. Hum. Reprod..

[30] Mantovani A, Sica A (2010). Macrophages, innate immunity and cancer: balance, tolerance, and diversity. Curr. Opin. Immunol..

[31] Sica A (2008). Macrophage polarization in tumour progression. Semin. Cancer Biol..

[32] Cupurdija K (2004). Macrophages of human first trimester decidua express markers associated to alternative activation. Am. J. Reprod. Immunol..

[33] Heikkinen J, Mottonen M, Komi J, Alanen A, Lassila O (2003). Phenotypic characterization of human decidual macrophages. Clin. Exp. Immunol..

[34] Nagamatsu T, Schust DJ (2010). The immunomodulatory roles of macrophages at the maternal-fetal interface. Reprod. Sci..

[35] Renaud SJ, Graham CH (2008). The role of macrophages in utero-placental interactions during normal and pathological pregnancy. Immunol. Invest..

[36] Svensson-Arvelund J (2015). The human fetal placenta promotes tolerance against the semiallogeneic fetus by inducing regulatory T cells and homeostatic M2 macrophages. J. Immunol..

[37] Le Bouteiller P, Tabiasco J (2006). Killers become builders during pregnancy. Nat. Med..

[38] Ashkar AA, Di Santo JP, Croy BA (2000). Interferon gamma contributes to initiation of uterine vascular modification, decidual integrity, and uterine natural killer cell maturation during normal murine pregnancy. J. Exp. Med..

[39] Gerosa F (2002). Reciprocal activating interaction between natural killer cells and dendritic cells. J. Exp. Med..

[40] Nedvetzki S (2007). Reciprocal regulation of human natural killer cells and macrophages associated with distinct immune synapses. Blood.

[41] Lapaque N, Walzer T, Meresse S, Vivier E, Trowsdale J (2009). Interactions between human NK cells and macrophages in response to *Salmonella* infection. J. Immunol..

[42] Kammerer U (2003). Unique appearance of proliferating antigen-presenting cells expressing DC-SIGN (CD209) in the decidua of early human pregnancy. Am. J. Pathol..

[43] Vacca P (2006). Analysis of natural killer cells isolated from human decidua: evidence that 2B4 (CD244) functions as an inhibitory receptor and blocks NK-cell function. Blood.

[44] Vacca P (2010). Crosstalk between decidual NK and CD14(+) myelomonocytic cells results in induction of Tregs and Immuno-suppression. Proc. Natl Acad. Sci. USA.

[45] Quack KC, Vassiliadou N, Pudney J, Anderson DJ, Hill JA (2001). Leukocyte activation in the decidua of chromosomally normal and abnormal fetuses from women with recurrent abortion. Hum. Reprod..

[46] Cooper MA (2002). In vivo evidence for a dependence on interleukin 15 for survival of natural killer cells. Blood.

[47] Choi SS (2004). Interleukin-15 enhances cytotoxicity, receptor expression, and expansion of neonatal natural killer cells in long-term culture. Clin. Diagn. Lab. Immunol..

[48] Hanna J (2003). CXCL12 expression by invasive trophoblasts induces the specific migration of CD16-human natural killer cells. Blood.

[49] Manaster I (2008). Endometrial NK cells are special immature cells that await pregnancy. J. Immunol..

[50] Hoxie JA (1993). Internalization and recycling of activated thrombin receptors. J. Biol. Chem..

[51] Verma S, Hiby SE, Loke YW, King A (2000). Human decidual natural killer cells express the receptor for and respond to the cytokine interleukin 15. Biol. Reprod..

[52] Strbo N (2006). Short-term cytolytic mediators’ expression in decidual lymphocytes is enhanced by interleukin-15. Am. J. Reprod. Immunol..

