# Supplementary figures and images for: Trophoblast-derived CXCL16 induces M2 macrophage polarization that in turn inactivates NK cells at the maternal–fetal interface
Source: Cell Mol Immunol. 2018 Mar 27;15(12):1038–46. doi: 10.1038/s41423-018-0019-x (PMC6269500; doi:10.1038/s41423-018-0019-x)

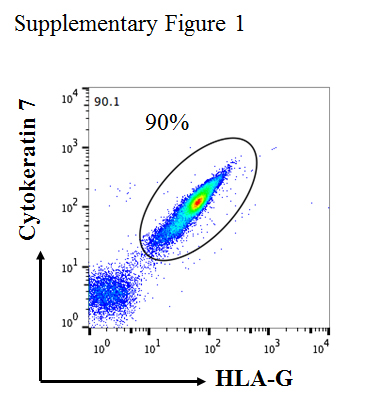

Supplement: Supplementary file 1 — Supplementary Figure 1 [file 41423_2018_19_MOESM1_ESM.jpg]

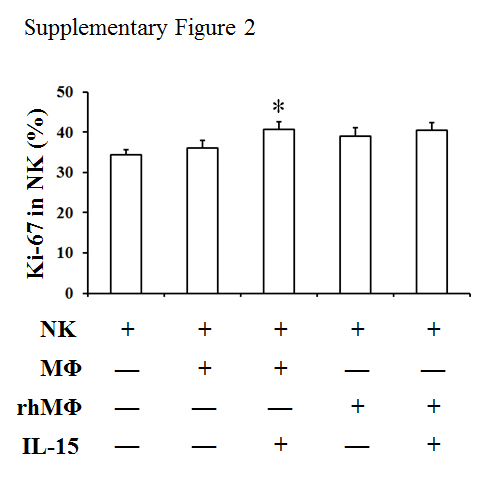

Supplement: Supplementary file 2 — Supplementary Figure 2 [file 41423_2018_19_MOESM2_ESM.jpg]
